# Supplementary material for: Polymorphisms in Pfkelch13 domains before and after the introduction of artemisinin-based combination therapy in Southwest Nigeria
Source: PLoS One. 2025 Mar 31;20(3):e0316479. doi: 10.1371/journal.pone.0316479 (PMC11957316; doi:10.1371/journal.pone.0316479)
Supplement: Supporting information 2 — (ZIP) [file pone.0316479.s002.zip › 050KNIFW_PREMIX_Plate_CORKELCH_G01.pdf]

Samples: 12262
Bases: 831
Average spacing: 15

8/17/2022

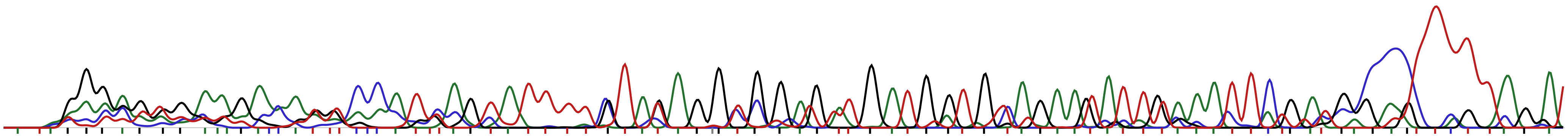

10 20 30 40 50 60 70 80 90

A TA G G G A G G G A A A G A C C A T T T C C C A T T T A G T A T T T T G T A T A G -G T G G A G T T G A T G G T G T A G A A T A T T T A A A T T C T G A T G A G A G T T C T T A G A

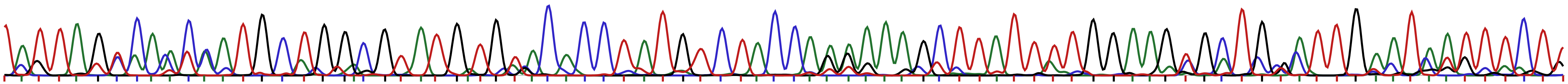

100 110 120 130 140 150 160 170

T A T T A G C C A A C A A T G C T G G A C G T A T G A T G T A C A C C T A T G T C T A C C A A A A A A G C T T A T T T T G G A A G T G C T G T A T T G A A T A A T T T C T T

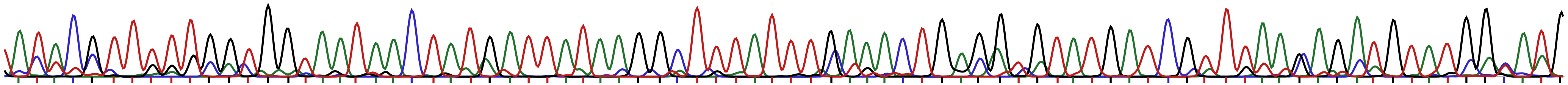

180 190 200 210 220 230 240 250 260

A T A C G T T T T T G G T G G T A A T A A C T A T G A T T A T A A G G C T T T A T T T G A A A C T G A G G T G T A T G A T C G T T T A A G A G A T G T A T G G C A T G

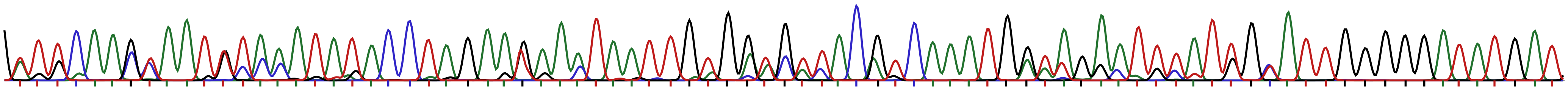

270 280 290 300 310 320 330 340

T T T C A A G T A A T T T A A A T A T A C C T A G A A G A A A T A A T T G T G G T G T T A C G T C A A A T G G T A G A A T T T A T T G C A T T G G G G G A T A T G A T

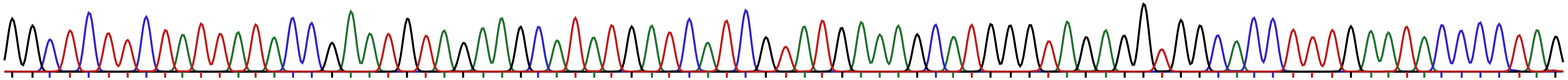

350 360 370 380 390 400 410 420

G G C T C T T C T A T T A T A C C G A A T G T A G A A G C A T A T G A T C A T C G T A T G A A A G C A T G G G T A G A G G T G G C A C C T T T G A A T A C C C C T A G

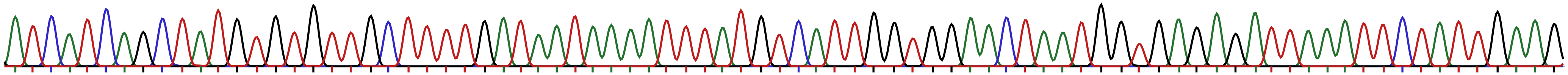

430 440 450 460 470 480 490 500

A T C A T C A G C T A T G T G T G T T G C T T T T G A T A A T A A A A T T T A T G T C A T T G G T G G A A C T A A T G G T G A G A G A T T A A A T T C T A T T G A A G

Samples: 12262
Bases: 831
Average spacing: 15

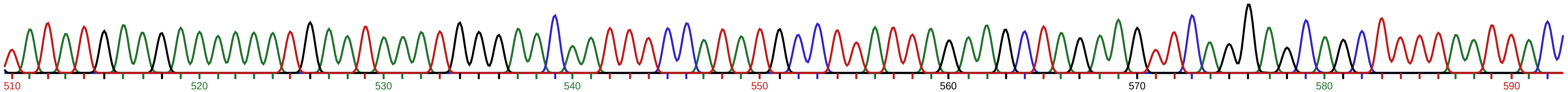

510 520 530 540 550 560 570 580 590

TATATGAAGAAAAAATGAATAAATGGGAACAATTTCCATATGCCTTATTAGAAGCTAGAAGTTCAGGAGCAGCTTTTAATTAC

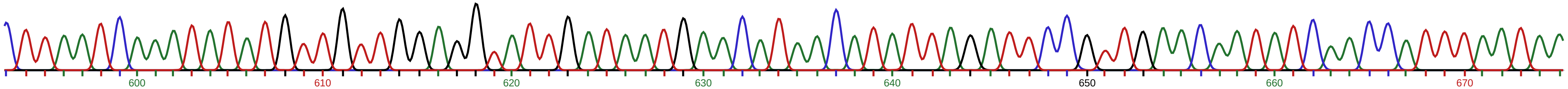

600 610 620 630 640 650 660 670

CTTAATCAAATATATGTTGTTGGAGGTATTGATAATGAACATAACATATTAGATTCCGTTGAACAATATCAACCATTTAATAA

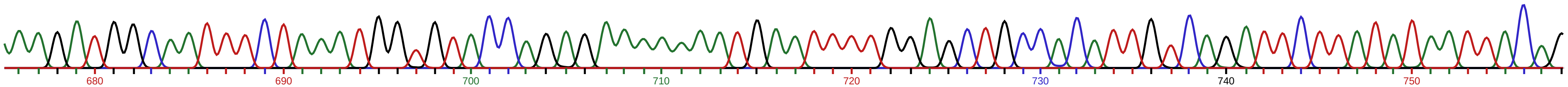

680 690 700 710 720 730 740 750

AAGATGGCAATTTCTAAATGGTGTACCAGAGAAAAAAATGAATTTTGGAGCTGCCACATTGTCAGATTCTTATATAATTACAG

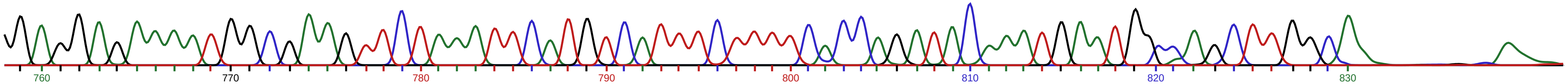

760 770 780 790 800 810 820 830

GAGGAGAAAATGGCGAAGTTCTAAATTCATGTCATTTCTTTTCACCAGATACAAATGAATGCCAGCTTGGCA

Samples: 12262
Bases: 831
Average spacing: 15
